# Genetic Dissection of Strain Dependent Paraquat-induced Neurodegeneration in the Substantia Nigra Pars Compacta

**DOI:** 10.1371/journal.pone.0029447

**Published:** 2012-01-24

**Authors:** Yun Jiao, Lu Lu, Robert W. Williams, Richard J. Smeyne

**Affiliations:** 1 Department of Developmental Neurobiology, Saint Jude Children's Research Hospital, Memphis, Tennessee, United States of America; 2 Department of Anatomy and Neurobiology, Center for Integrative and Translational Genomics, University of Tennessee Health Science Center, Memphis, Tennessee, United States of America; University of Nebraska Medical Center, United States of America

## Abstract

The etiology of the vast majority of Parkinson's disease (PD) cases is unknown. It is generally accepted that there is an interaction between exposures to environmental agents with underlying genetic sensitivity. Recent epidemiological studies have shown that people living in agricultural communities have an increased risk of PD. Within these communities, paraquat (PQ) is one of the most utilized herbicides. PQ acts as a direct redox cycling agent to induce formation of free radicals and when administered to mice induces the cardinal symptoms of parkinsonism, including loss of TH+-positive dopaminergic (DA) neurons in the ventral midbrain's substantia nigra pars compacta (SNpc). Here we show that PQ-induced SNpc neuron loss is highly dependent on genetic background: C57BL/6J mice rapidly lose ∼50% of their SNpc DA neurons, whereas inbred Swiss-Webster (SWR/J) mice do not show any significant loss. We intercrossed these two strains to map quantitative trait loci (QTLs) that underlie PQ-induced SNpc neuron loss. Using genome-wide linkage analysis we detected two significant QTLs. The first is located on chromosome 5 (Chr 5) centered near *D5Mit338*, whereas the second is on Chr 14 centered near *D14Mit206*. These two QTLs map to different loci than a previously identified QTL (*Mptp1*) that controls a significant portion of strain sensitivity to 1-methyl-4-phenyl-1,2,3,6-tetrahydropyridine (MPTP), suggesting that the mechanism of action of these two parkinsonian neurotoxins are different.

## Introduction

Parkinson's disease (PD) is the third most common neurodegenerative disorder, affecting approximately two percent of the adult population older than 55 years. The underlying cause for the vast majority of PD cases is unknown. Controversy still exists as to how much of the disease results from strictly genetic factors, environmental factors, or an interaction of both [1], [2], [3]. Empirical evidence suggests that less than 10% of all diagnosed Parkinsonism has a strict familial etiology [4]. One mechanism related to environmental exposure that has been proposed in PD's etiology is the abnormal handling of free radical species; whether by excessive generation of these species or inability to handle their detoxification [5], [6]. Several animal models of PD that utilize xenobiotics have been developed; each mimicking aspects of parkinsonism. These include administration of 1-methyl-4-phenyl-1,2,3,6-tetrahydropyridine (MPTP), rotenone or 1,1′ di methyl-4,4′-bipyridium dichloride (paraquat, PQ). Each of these toxins generates free radicals, although the mechanism(s) by which this occurs is different. MPTP and rotenone generate oxidative stress through generation of free radicals after blockade of complex 1 in the mitochondrial electron transport chain [7]. Paraquat also generates free radicals, but through direct redox cycling [8], [9].

Only specific strains of mice are sensitive to the administration of MPTP [10], [11], [12]. The differential effects of xenobiotics on CNS, including environmental and chemical toxins such as MPTP and PQ, are likely influenced by the interaction of multiple gene products. The cumulative phenotypes that arise from both environmental factors and polygenic interactions among gene variants are termed quantitative traits. Chromosomal regions that harbor crucial gene variants that modulate risk are called quantitative trait loci (QTLs) [13]. The premise behind QTL mapping is that if numerous genetic markers are examined, only those that cosegregate with a particular phenotype variant, for example, high or low susceptibility, will be linked to the gene variants that underlie that trait [14]. Previously, we have shown that the effects of MPTP on SNpc neuron loss are strain specific. The very well characterized C57BL/6J strain is highly sensitive to this compound whereas the common Swiss–Webster inbred strain (SWR/J) is resistant. Through a genome-wide analysis of C57BL/6J×SWR backcross progeny we mapped a QTL for MPTP-induced SNpc neuron loss named *Mptp1* near the distal end of Chr 1 between *D1Mit113* and *D1Mit293*. This locus accounts for the majority of the strain sensitivity to MPTP [15]. In order to determine if genes involved in controlling DA neuron loss are similar in MPTP and PQ-induced neurodegeneration, we identified QTLs for PQ loss again exploiting the same strain difference. Our findings suggest that there are also marked strain-specific effects of PQ on SNpc dopaminergic neuron number loss, and that the genetic influence underlying PQ-induced neurons loss are different than those seen with MPTP.

## Materials and Methods

All of the experimental procedures in the animals were performed in accordance with the NIH Guide for the Care and Use of Laboratory Animals and all protocols, were approved by the St Jude Children's Research Hospital IACUC (protocol 270). Experiments were carried out in accordance with The Code of Ethics of the World Medical Association (Declaration of Helsinki) for animal experiments.

Male and female C57BL/6J and SWR/J mice were purchased from the Jackson Laboratory (Bar Harbor, ME). F1 crosses were generated by mating male C57BL/6J with female SWR/J and female C57BL/6J with male SWR/J stock. F1 hybrids were backcrossed to SWR/J to generate a set of 61 backcross (N2) progeny that were used to map QTLs. All animals were housed within the vivarium at St. Jude Children's Research Hospital and were maintained on a 12∶12 hour light∶dark cycle with *ad libitum* food and water.

### Paraquat treatment

1,1′ di methyl-4,4′-bipyridium dichloride (paraquat, PQ) (catalog 36541 Sigma-St. Louis, MO) was dissolved in sterile saline to a final concentration of 20 mg/ml. Each animal was given a total of 60 mg/kg of PQ, using a dosage regimen of 10 mg/kg×2 per week for 3 weeks. All mice that survived the injection protocol were sacrificed one week after the final PQ administration.

### Histology

Mice were anesthetized with an overdose of Avertin. Following induction of deep anesthesia determined by loss of deep tendon and corneal reflexes, animals were transcardially perfused with physiologic saline followed by 3% paraformaldehyde in 1X phosphate-buffered saline (PBS), pH 7.4. Brains were removed from the calvaria and post-fixed overnight in fresh fixative, dehydrated through a graded series of ethanols, defatted in mixed xylenes and embedded in Paraplast-X-tra (Fisher Scientific, Pittsburgh, PA). Brains were subsequently blocked and serially sectioned at 10 microns in the coronal plane. All sections from the rostral hippocampus to the cerebellar-midbrain junction was saved and mounted onto Superfrost-Plus slides (Fisher Scientific).

Standard immunhistochemical techniques using a polyclonal antibody directed against tyrosine hydroxylase (TH) (1∶250 in blocking buffer; Pel Freez, Rogers, AR) were to identify dopaminergic neurons in the SNpc as previously described [15]. Slides were then counterstained with Neutral Red, dehydrated through a graded series of alcohol, mounted in Permount and coverslipped.

### DA Cell Quantification and Analysis

Dopaminergic neurons in the SNpc were quantified using stereological methods described previously [16]. Statistical analyses were done using Student's *t*-test (GraphPad Prism V, La Jolla, CA).

### Microsatellite markers

To identify and map QTLs, we used a set of polymorphic MIT microsatellite markers (Table 1) that previously have been shown to differentiate C57BL/6J from SWR/J [15]. DNA samples from each mouse were amplified using PCR thermal cycling parameters described in detail at www.nervenet.org/papers/PCR.html. We used a touchdown PCR protocol to improve the specificity of annealing. The products were all run on Metaphor agarose, photographed, and scored manually. Data were entered into a relational database (FileMaker Pro).

**Table 1:** List of Microsatellite Markers and Mendelian Correlations.

MIT marker	cM	Mendelian Correlation	MIT marker	cM	Mendelian Correlation
D1Mit211	10.59	0.013	D11Mit78	10.44	0.247
D1Mit100	62.56	0.023	D11Mit5	40.59	0.142
D1Mit293	97.55	0.064	D11Mit334	74.06	0.169
D2Mit416	12.00	0.009	D12Mit169	7.03	0.352
D2Mit458	29.62	0.092	D12Mit214	37.86	0.253
D2Mit311	86.12	0.069	D12Mit280	60.94	0.392
D3Mit240	15.80	0.221	D13Mit106	47.75	0.362
D3Mit51	26.20	0.253	D13Mit254	40.95	0.392
D3Mit19	66.70	0.198	D13Mit78	67.21	0.253
D4Mit192	13.50	0.299	**D14Mit206**	**11.53**	**0.429**
D4Mit78	61.15	0.197	D14Mit262	37.20	0.247
D4Mit13	75.67	0.121	D14Mit266	64.86	0.090
D5Mit233	28.55	0.250	D15Mit53	6.29	.0256
**D5Mit338**	**52.23**	**0.428**	D15Mit229	16.31	0.310
D5Mit287	89.18	0.325	D15Mit161	52.78	0.203
D6Mit273	22.51	0.143	D16Mit181	2.90	0.066
D6Mit146	43.05	0.101	D16Mit4	25.43	0.034
D6Mit291	66.78	0.169	D16Mit106	57.68	0.067
D7Mit117	17.26	0.041	D17Mit30	14.26	0.037
D7Mit238	63.78	0.169	D17Mit139	27.40	0.352
D7Mit259	88.85	0.250	D17Mit42	50.30	0.066
D8Mit95	12.47	0.172	D18Mit223	6.60	0.037
D8Mit205	28.85	0.218	D18Mit188	45.88	0.253
D8Mit121	72.27	0.170	D18Mit213	57.33	0.351
D9Mit205	20.75	0.168	D19Mit90	35.97	0.233
D9Mit32	36.41	0.196	D19Mit137	54.60	0.221
D9Mit116	59.58	0.305	DXMit166	28.26	0.175
D10Mit247	5.81	0.231	DXMit68	29.49	0.260
D10Mit186	38.56	0.325	DXMit117	53.75	0.314
D10Mit292	55.33	0.196			
D10Mit297	72.31	0.037			

### Calculating linkage between loci

We compared the distribution pattern of phenotypes of the mice (high or low SNpc number following PQ treatment) with the distribution pattern of sensitive (C57BL/6J = B) and resistant (SWR/J = S) alleles at each of the polymorphic microsatellite loci. The first level of analysis was simply to detect a linkage using a constrained additive regression model whereas the second level involved estimating QTL position more precisely by interval mapping. Actual calculations were performed using the program Map Manager QTX b29 [17].

### Microarray Analysis

Four- to six-month old C57BL/6J and SWR/J mice were deeply anesthesized, and when deep tendon and corneal reflexes they were absent rapidly decapitated. The substantia nigra (Bregma: −2.70 to −3.70) and striatum (Bregma: +0.14 to +1.26 mm) [18] were rapidly dissected, flash frozen and stored at −80°C. mRNA was isolated from SN and striatum in accordance with the protocol outlined in RNAqueous Micro kit (Ambion, Austin, TX) according to manufacturers recommendations. Technical procedures for microarray analysis, including quality control of mRNA, labeling, hybridization and scanning of the arrays were performed by the Hartwell Center for Bioinformatics & Biotechnology (HC) at St. Jude Children's Research Hospital (SJCRH) according to standard operating procedures for Affymetrix protocols (GeneChip Expression Analysis manual, Affymetrix, Santa Clara CA, USA).

The GeneChip HT MG-430 PM array plate (Affymetrix) containing 45,037 probe sets were used in this study. These arrays represent 39,000 transcripts. Scanned images of processed arrays were analyzed with the Gene Chip Operating Software (GCOSv1.2, Affymetrix). Assessment of probe set present/absent calls was made using the Single Array Analysis method using the statistical algorithm with default analysis parameters as detailed in http://media.affymetrix.com/support/technical/whitepapers/ht_system_whitepaper.pdf. Expression levels of genes located within the QTL regions were queried and those having a difference in expression (±25%) and a significance of p<0.001 were identified.

## Results

### Strain specific Effects of PQ on SNPC dopaminergic neuron number

**Figure 1:**
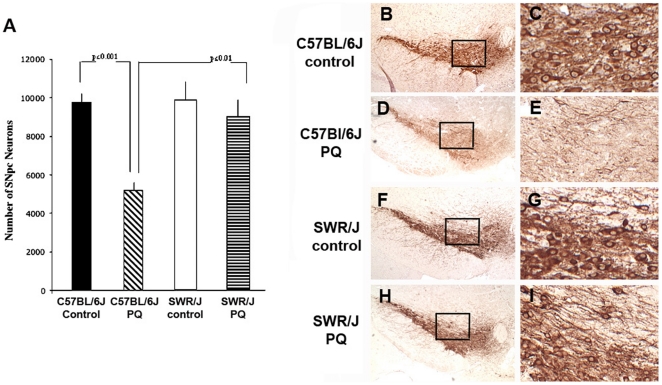
Paraquat-induced cell loss is strain dependent. (A) After chronic PQ administration, we find that C57BL/6J have a ≈50% decrease in SNpc DA neurons, while SWR have a ≈10% loss of SNpc neurons. (B) TH-immunostained section through rostral SNpc of C57BL/6J mouse. Box is seen at higher magnification in (C). (D) Following chronic administration of PQ to C57BL/6J mice, there is a loss of both DA neurons and fibers. Box is seen at higher magnification in (E). (F). TH-immunostained section through rostral SNpc of SWR mouse. Box is seen at higher magnification in (G). (H) Following chronic administration of PQ to SWR mice, there is a no apparent loss of DA neurons or fibers. Box is seen at higher magnification in (E).

We compared the effects of PQ on the severity of SNpc dopaminergic neuron loss in C57BL/6J and SWR/J strains. We have previously shown that these strains were respectively susceptible and resistant to MPTP-induced SNpc dopamine neuron loss [19]. We find that there is a strain-dependent sensitivity to PQ-induced SNpc neuron loss, and that C57BL/6J lose ∼48% of SNpc dopaminergic neurons, whereas SWR/J show a much less and insignificant 8% loss (Figure 1).

### Identification of QTL's underlying strain dependent PQ –induced SNpc neuron loss

**Figure 2:**
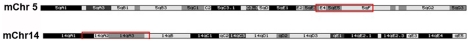
Chromosomal maps showing the identified QTL regions. A 20 cM region (red box) on the distal are of mCHr 5 and the proximal arm of mChr14 were identified by QTL analysis.

To identify quantitative trait loci responsible for PQ-induced strain dependent SNpc DA neuron loss, we used stereological procedures to estimate the number of SNpc DA neurons in 61 C57BL/6J×SWR/J N2 progeny. Only C57BL/6J×SWR/J F1 mice are sensitive to PQ, and we therefore crossed C57BL/6J×SWR/J F1 to SWR/J males to generate the N2 backcross progeny that will be B/S heterozygotes or S/S homozygous at each locus. Genotypes were entered for each marker and correlated to two phenotypes: (1) the overall phenotypic severity treated as a Mendelian score (affected or unaffected), and (2) the number of SNpc DA neurons [15]. We found two significant (genome-wide P<0.001) chromosomal loci using the Mendelian correlation with a 20 cM sensitivity, and the program Map Manager QTX b29 based on 10,000 permutation analysis, located on Chr 5 centered near D5Mit338 (Chr5, ≈109 Mb, all genome positions are based on the NCBI37/mm9 mouse genome assembly) (Fig. 2A) and on Chr 14 centered near D14Mit206 (Chr 14, ≈21.5 Mb) (Figure 2B)(Table 1). The same loci were found using the numerical data.

The QTL on Chr 5 overlaps 88 coding genes whereas that on Chr 14 overlaps 28 coding genes (as delineated by Ensembl). Based on our analysis of array data, the majority of these genes (93 of 116) have detectible expression in either striatum or substantia nigra in C57BL/6J and SWR/J. Of 88 identified genes located on Chr 5 between 99 and 119 Mb, 31 were differentially expressed by more than 25% between strains (p<0.001, Table S1) in striatum. Only five genes met the same criterion on the Chr 14 interval between 16.4 and 26.6 Mb (Table S2). In addition to the identified coding genes, the Ensembl database also identified a sequence on Chr 5 between 117.082490 and 117.083614 Mb. that corresponds to a glutathione S-transferase Mu pseudogene (*GSTm2-ps1*).

## Discussion

In this study we report that the C57BL/6J and SWR/J strains are differentially sensitive to systemic administration of paraquat—a finding that supports previous studies that detail differential genetic effects of this herbicide [20] and other neurotoxins, including MPTP [12], [19], [21], [22], [23]. We have exploited this pronounced strain difference to map chromosomal regions that modulate the differential vulnerability of dopaminergic cell to PQ. Unlike studies of Parkinson's disease in humans, we can carefully control both genetic and environmental factors and efficiently generate precise estimates of the loss of DA neurons in inbred parental strains and backcross progeny. As shown here, the parental strain difference can be dissected into a small number of QTLs and candidate genes.

PQ is one of the most commonly used pesticides in the agricultural community. Its mechanism of action involves the transfer of an electron (usually from NADPH) to form a PQ+ radical. This free radical interacts with molecular oxygen to form a superoxide radical that damages lipids contained within cell membranes [24]. PQ has been shown to induce extensive mitochondrial oxidative damage [25], [26]. In the brain, PQ is actively transported through neutral amino acid transporters [27] and its use has been linked to an increased risk for developing Parkinson's disease [28], [29], [30]. Experimentally, systemic administration of paraquat induces a relatively specific lesion in the SNpc that results in dopaminergic neuron loss [20], [31], [32]. Mechanistically, it has been proposed that this selective cell loss occurs by virtue of the SNpc having 1) significant dopamine metabolism [33], 2) a significantly increased microglial density compared to other brain regions [34], and 3) an increased concentration of iron which results in a propensity to form intracellular hydrogen peroxide and superoxides [35] that can initiate apoptosis through a BAK dependent mechanism [36]. Although these cellular mechanisms have been hypothesized, the gene(s) underlying them have not been identified.

Our analysis identified two QTLs for PQ senstivity; one located within a 20 cM (100∼120 Mb) interval of Chr 5 and the other within a 20 cM (15∼35 Mb) interval of Chr 14. To better define genes in these regions that may contribute to differential sensitivity to PQ-induced SNpc neuronal loss we used several criteria. First, we used an unbiased approach to identify genes that are differentially expressed in the substantia nigra and striatum of the parental strains. Although a number of genes meet our criteria of a 25% difference in expression, we further filtered results based upon the known function of the genes and possible relations to a function that could modulate PQ effects. We also flagged any candidates identified in previous genome-wide association studies of humans. The latter approach highlighted diacylglycerol kinase, theta 110 kDa (DGKQ), a gene that has a strong association in patients of Dutch decent with familial [37] and sporadic [38] Parkinson's disease Inhibition of DGKQ activity attenuates the binding of SF1 to the *CYP17* promoter, subsequently inhibiting cAMP-dependent *CYP17* transcription. CYP17 is a member of the P450 proteins that function as xenobiotic metabolizing enzymes [39], which act in the modulation of free radicals in the nervous system [40]. Other genes within the QTL were implicated by their known function; where modulation of these activities have been implicated in the pathogenesis of Parkinson's disease. Examples of these genes include *Spp1* and *Hspb8*, each of which has been implicated in the inflammatory, oxidative, and nitrosylative stress response to insult [41]. *Spp1* encodes the osteopontin protein that is expressed in the SNpc [42] and its absence has been shown to be neuroprotective in the MPTP model of experimental parkinsonism [43]. *Hspb8* encodes a heat shock protein that forms a complex BAG3 [44]. When overexpressed, this HSPB8-BAG3 complex functions in the clearance of mutated aggregation-prone proteins including alpha-synuclein [45], whose accumulation is a hallmark of Parkinson's disease [46].

Other genes in these QTLs function in processes thought to be important to neuronal survival following injury. There is higher expression in genes involved in energy production and gluconeogenesis in the SN, where their gene products function to increase production of ATP, and indirectly (*Adk*) or directly (*Hscb*) contribute to protection from oxidative stress [44], [47], [48]—a critical process in SNpc DA neuroprotection [5], [49], [50]. Additionally, *Ppp3cb*, which encodes a subunit the calcineurin, is a protein that is highly expressed in the SN [51] and functions as a phosphatase that modulates synaptic plasticity and cell death [52], [53]. Lower levels of the citron mRNA are seen in both SN and striatum of SWR mice exposed to PQ compared to C57BL/6J. Citron acts as a rho/rac binding protein that regulates activity of RhoA. Inhibition of RhoA is associated with repair of axonal processes [54] and has been shown to increase its expression in brain after treatment with the complex I inhibitor rotenone [55]. Conversely, citron levels are reduced in animals exposed to environmental enrichment [56], which has been shown to be neuroprotective [57].

A third class of genes differentially expressed between C57BL/6J and SWR/J following PQ administration are in the inflammatory pathway. *Nos1*, a gene encoding neuronal nitric oxide synthase, functions to catalyze the production of nitric oxide (NO) from L-arginine. Inhibition of nNOS in neuronal cells lines increases the toxicity of MPP+ [58], suggesting that the higher mRNA expression levels seen in the SWR striatum would be neuroprotective.

We also noted the presence of sequence within the Chr 5 QTL that encodes a sequence that appears to be glutathione S-transferase mu (*Gstm1*) pseudogene. Pseudogenes resemble their cognate genes, but for the most part are not translated into functional proteins. Although they often lack introns, likely as a function of their generation through retrotransposition, these sequences are not likely to be “junk DNA” as previously thought [59]. Recent evidence suggests that they can play a role in the regulation of their related coded gene [60], [61], [62]. *Gstm1* is a member of the GST superfamily, that function as phase II detoxification enzymes that catalyze the conjugation of glutathione and electrophiles [63]. *Gstm1* is one of seven members in a closely associated gene cluster located on mouse Chr3 [64]. *Gstm1* is expressed in brain [65], and in the substantia nigra is seen in both dopaminergic neurons and astrocytes [66] and has been implication in control of dopamine metabolism [67] that could have implications in the etiology of Parkinson's disease.

In a previous QTL examining sensitivity to the parkinsonian agent MPTP, we identified a single QTL called *Mptp1*, located on Chr1 [15] and a strong candidate gene, glutathione S-transferase pi (*Gstp1*). *Gstp1* is a member of the same GST superfamily as *Gstm1* and also functions as a phase II detoxification enzyme. The location of the two significant QTLs in this study do not map to the *Mptp1* QTL, although the same two mouse strains show a similar phenotype after treatment with MPTP and PQ including loss of DA neurons and induction of micro-and astrogliosis. This difference is, however, not unexpected since the mechanism of action of MPTP, which involves blockade of complex I of the electron transport chain leading to generation of free radicals [68], has been shown to be different from PQ, which is a direct redox generator [8]. It would be interesting to determine if the effects of these two xenobiotics are synergistic, suggesting that there are independent populations of SNpc dopamine neurons that are sensitive to different xenobiotic insults and thus different interventions would be necessary for each xenobiotic. However, if PQ, MPTP or other exogenous agents (i.e. rotenone) kill the same populations of SNpc dopamine neurons, independent of the mechanism that initiates the cell death, one could concentrate on developing a general therapy to reduce oxidative stress in the brain as a method for protecting against or slowing the progress of the SNpc dopamine neuron death.

## Supporting Information

Table S1 **Differential Gene Expression in Substantia Nigra and Striatum on mChr 5.**
(DOCX)

Table S2 **Differential Gene Expression in Substantia Nigra and Striatum on mChr 14.**
(DOCX)
