# Supplementary material for: Genetic Dissection of Strain Dependent Paraquat-induced Neurodegeneration in the Substantia Nigra Pars Compacta
Source: PLoS One. 2012 Jan 24;7(1):e29447. doi: 10.1371/journal.pone.0029447 (PMC3265472; doi:10.1371/journal.pone.0029447)
Supplement: Table S1 — Differential Gene Expression in Substantia Nigra and Striatum on mChr 5. (DOCX) [file pone.0029447.s001.docx]

Table S1

Differential Gene Expression in Substantia Nigra and Striatum on mChr 5

| **Gene Name** | **Gene Symbol** | **SWR/C57**  **SN** | **SWR/C57**  **striatum** |
| --- | --- | --- | --- |
| adrenergic receptor kinase, beta 2 | Adrbk2 | 0.79 | 0.98 |
| AF4/FMR2 family, member 1 | Aff1 | 0.93 | 0.95 |
| **ankyrin repeat domain 13a** | **Ankrd13a** | **0.67** | 1.00 |
| **aspartate beta-hydroxylase domain containing 2** | **Asphd2** | **0.72** | 1.14 |
| ATP synthase, H+ transporting, mitochondrial F1F0 complex, subunit e | Atp5k | 1.10 | 0.98 |
| **bromodomain, testis-specific** | **Brdt** | 0.97 | **1.88** |
| coiled-coil domain containing 18 | Ccdc18 | 1.16 | 0.94 |
| coiled-coil domain containing 64 | Ccdc64 | 0.87 | 1.18 |
| CDP-diacylglycerol synthase 1 | Cds1 | 0.90 | 1.04 |
| **checkpoint with forkhead and ring finger domains** | **Chfr** | **1.25** | 1.23 |
| **citron** | **Cit** | **0.38** | **0.58** |
| COP9 (constitutive photomorphogenic) homolog, subunit 4 (Arabidopsis thaliana) | Cops4 | 1.03 | 0.96 |
| coenzyme Q2 homolog, prenyltransferase (yeast) | Coq2 | 0.79 | 0.91 |
| **coenzyme Q5 homolog, methyltransferase (yeast)** | **Coq5** | **1.31** | **1.37** |
| coronin, actin binding protein 1C | Coro1c | 0.89 | 0.69 |
| complexin 1 | Cplx1 | 0.97 | 1.16 |
| DEAD (Asp-Glu-Ala-Asp) box polypeptide 51 | Ddx51 | 0.91 | 1.15 |
| **diacylglycerol kinase, theta** | **Dgkq** | **0.64** | **1.40** |
| down-regulator of transcription 1 | Dr1 | 0.87 | 1.16 |
| dynein light chain LC8-type 1 | Dynll1 | 1.09 | 0.99 |
| enolase-phosphatase 1 | Enoph1 | 0.81 | 0.93 |
| ecotropic viral integration site 5 | Evi5 | 1.04 | 1.03 |
| **F-box protein 21** | **Fbxo21** | **0.75** | 0.87 |
| F-box and WD-40 domain protein 8 | Fbxw8 | 0.81 | 1.24 |
| cyclin G associated kinase | Gak | 0.90 | 1.01 |
| **UDP-N-acetyl-alpha-D-galactosamine:polypeptide N-acetylgalactosaminyltransferase 9** | **Galnt9** | **0.50** | **0.57** |
| **GCN1 general control of amino-acid synthesis 1-like 1 (yeast)** | **Gcn1l1** | **0.66** | 0.89 |
| **G protein-coupled receptor kinase-interactor 2** | **Git2** | **0.59** | 0.90 |
| **glomulin, FKBP associated protein** | **Glmn** | **1.26** | 1.16 |
| golgi autoantigen, golgin subfamily a, 3 | Golga3 | 1.02 | 0.81 |
| heterogeneous nuclear ribonucleoprotein D-like | Hnrpdl | 1.17 | 0.96 |
| Hermansky-Pudlak syndrome 4 homolog (human) | Hps4 | 1.21 | 1.14 |
| **HscB iron-sulfur cluster co-chaperone homolog (E. coli)** | **Hscb** | **1.36** | 1.03 |
| **heat shock protein 8** | **Hspb8** | **0.75** | **0.62** |
| **iduronidase, alpha-L-** | **Idua** | **0.62** | 0.98 |
| IscU iron-sulfur cluster scaffold homolog (E. coli) | Iscu | 1.01 | 1.11 |
| potassium channel tetramerisation domain containing 10 | Kctd10 | 1.01 | 0.95 |
| kelch-like 8 (Drosophila) | Klhl8 | 0.97 | 0.95 |
| lin-54 homolog (C. elegans) | Lin54 | 1.23 | 0.91 |
| **leucine rich repeat containing 8 family, member C** | **Lrrc8c** | **1.28** | 0.94 |
| mitogen-activated protein kinase 10 | Mapk10 | 1.02 | 0.96 |
| mediator complex subunit 13-like | Med13l | 0.93 | 1.12 |
| methylmalonic aciduria (cobalamin deficiency) type B homolog (human) | Mmab | 1.09 | 0.77 |
| meningioma 1 | Mn1 | 0.99 | 1.21 |
| **mitochondrial ribosomal protein S18C** | **Mrps18c** | **1.33** | 0.92 |
| **metal response element binding transcription factor 2** | **Mtf2** | **1.28** | 1.03 |
| nucleolar complex associated 4 homolog (S. cerevisiae) | Noc4l | 0.99 | 0.89 |
| **nitric oxide synthase 1, neuronal** | **Nos1** | 1.10 | **1.36** |
| nudix (nucleoside diphosphate linked moiety X)-type motif 9 | Nudt9 | 0.93 | 1.02 |
| polycomb group ring finger 3 | Pcgf3 | 1.15 | 1.04 |
| phosphoglycerate mutase family member 5 | Pgam5 | 0.95 | 1.09 |
| phosphatidylinositol glycan anchor biosynthesis, class G | Pigg | 0.95 | 0.88 |
| phosphatidylinositol transfer protein, beta | Pitpnb | 1.23 | 1.07 |
| polycystic kidney disease 2 | Pkd2 | 0.84 | 0.98 |
| **phosphatidylinositol-specific phospholipase C, X domain containing 1** | **Plcxd1** | **1.27** | 0.86 |
| protein kinase, AMP-activated, beta 1 non-catalytic subunit | Prkab1 | 1.02 | 1.11 |
| **protein kinase, cGMP-dependent, type II** | **Prkg2** | **1.31** | 1.16 |
| peroxisomal membrane protein 2 | Pxmp2 | 0.85 | 1.07 |
| **paxillin** | **Pxn** | **0.69** | 0.94 |
| RAB35, member RAS oncogene family | Rab35 | 0.70 | 0.95 |
| RasGEF domain family, member 1B | Rasgef1b | 1.15 | 1.10 |
| replication factor C (activator 1) 5 | Rfc5 | 1.22 | 1.11 |
| ring finger protein 10 | Rnf10 | 1.00 | 1.24 |
| RNA polymerase II associated protein 2 | Rpap2 | 1.14 | 0.92 |
| RNA polymerase II associated protein 2 | Rpap2 | 0.92 | 0.92 |
| squamous cell carcinoma antigen recognized by T-cells 3 | Sart3 | 0.99 | 1.03 |
| **seizure related 6 homolog like** | **Sez6l** | **0.70** | 1.26 |
| splicing factor, arginine/serine rich 9 | Sfrs9 | 1.04 | 1.00 |
| small G protein signaling modulator 1 | Sgsm1 | 0.76 | 1.02 |
| SPARC-like 1 | Sparcl1 | 1.18 | 1.05 |
| **secreted phosphoprotein 1** | **Spp1** | **0.63** | **0.51** |
| signal peptide peptidase 3 | Sppl3 | 0.81 | 1.07 |
| suppressor of defective silencing 3 homolog (S. cerevisiae) | Suds3 | 0.79 | 1.13 |
| SV2 related protein | Svop | 0.63 | 0.91 |
| TAO kinase 3 | Taok3 | 1.11 | 1.23 |
| trichoplein, keratin filament binding | Tchp | 0.79 | 1.50 |
| tuftelin interacting protein 11 | Tfip11 | 0.86 | 0.90 |
| transforming growth factor, beta receptor III | Tgfbr3 | 0.96 | 1.09 |
| **transmembrane emp24 protein transport domain containing 5** | **Tmed5** | **1.30** | 0.99 |
| **transmembrane protein 119** | **Tmem119** | 0.96 | **1.34** |
| protein-tyrosine sulfotransferase 2 | Tpst2 | 1.00 | 1.07 |
| TP53 regulated inhibitor of apoptosis 1 | Triap1 | 1.18 | 1.19 |
| ubiquitin protein ligase E3B | Ube3b | 0.76 | 1.17 |
| Unc-51 like kinase 1 (C. elegans) | Ulk1 | 0.80 | 1.12 |
| ubiquitin specific peptidase 30 | Usp30 | 1.03 | 1.04 |
| WD repeat and FYVE domain containing 3 | Wdfy3 | 1.10 | 1.11 |
| **WD repeat and SOCS box-containing 2** | **Wsb2** | **0.72** | 0.90 |
| **zinc finger protein 644** | **Zfp644** | **1.79** | **1.25** |
