# Supplementary material for: Genetic Dissection of Strain Dependent Paraquat-induced Neurodegeneration in the Substantia Nigra Pars Compacta
Source: PLoS One. 2012 Jan 24;7(1):e29447. doi: 10.1371/journal.pone.0029447 (PMC3265472; doi:10.1371/journal.pone.0029447)
Supplement: Table S2 — Differential Gene Expression in Substantia Nigra and Striatum on mChr 14. (DOCX) [file pone.0029447.s002.docx]

Table S2

Differential Gene Expression in Substantia Nigra and Striatum on mChr 14

| Gene name | Gene symbol | SWR/C57  SN | SWR/C57  striatum |
| --- | --- | --- | --- |
|  |  |  |  |
| **adenosine kinase** | **Adk** | **1.34** | 0.95 |
| annexin A7 | Anxa7 | 1.03 | 0.86 |
| adaptor-related protein complex 3, mu 1 subunit | Ap3m1 | 1.01 | 0.89 |
| coiled-coil-helix-coiled-coil-helix domain containing 1 | Chchd1 | 0.99 | 1.06 |
| catechol-O-methyltransferase domain containing 1 | Comtd1 | 0.76 | 1.11 |
| DnaJ (Hsp40) homolog, subfamily C, member 9 | Dnajc9 | 1.14 | 0.90 |
| ecdysoneless homolog (Drosophila) | Ecd | 0.96 | 0.97 |
| fucosyltransferase 11 | Fut11 | 1.10 | 1.18 |
| **guanine nucleotide binding protein (G protein), gamma 2** | **Gng2** | **1.45** | 0.95 |
| potassium large conductance calcium-activated channel, subfamily M, alpha member 1 | Kcnma1 | 0.96 | 1.01 |
| MYST histone acetyltransferase monocytic leukemia 4 | Myst4 | 0.99 | 1.18 |
| N-deacetylase/N-sulfotransferase (heparan glucosaminyl) 2 | Ndst2 | 0.90 | 0.90 |
| N-glycanase 1 | Ngly1 | 1.10 | 0.86 |
| nuclear receptor subfamily 1, group D, member 2 | Nr1d2 | 1.06 | 1.06 |
| nudix (nucleoside diphosphate linked moiety X)-type motif 13 | Nudt13 | 1.13 | 0.98 |
| 3-oxoacyl-ACP synthase, mitochondrial | Oxsm | 0.93 | 0.76 |
| **protein phosphatase 3, catalytic subunit, beta isoform** | **Ppp3cb** | **1.35** | 0.94 |
| retinoic acid receptor, beta | Rarb | 1.12 | 0.86 |
| ribosomal protein L15 | Rpl15 | 1.04 | 0.93 |
| sterile alpha motif domain containing 8 | Samd8 | 1.15 | 1.07 |
| Sec24 related gene family, member C (S. cerevisiae) | Sec24c | 0.93 | 1.06 |
| thyroid hormone receptor beta | Thrb | 0.96 | 1.12 |
| **topoisomerase (DNA) II beta** | **Top2b** | **1.64** | 0.87 |
| ubiquitin-conjugating enzyme E2E 2 (UBC4/5 homolog, yeast) | Ube2e2 | 0.92 | 1.06 |
| ubiquitin specific peptidase 54 | Usp54 | 0.84 | 0.82 |
| vinculin | Vcl | 1.10 | 1.10 |
| voltage-dependent anion channel 2 | Vdac2 | 1.20 | 0.92 |
| **zinc finger protein 503** | **Zfp503** | **.055** | 1.13 |
